# Evidence for Kidney Volume as a Measure of ADPKD Severity “Marches On” in the OVERTURE Study

**DOI:** 10.1016/j.ekir.2023.03.002

**Published:** 2023-03-11

**Authors:** Matthew B. Lanktree

**Affiliations:** 1 Departments of Medicine and Health Research Methodology, Evidence and Impact, Division of Nephrology, St. Joseph’s Healthcare Hamilton & McMaster University, Hamilton, Ontario, Canada

See Clinical Research on Page 989

In this issue of *Kidney International Reports,* Perrone *et al.*^1^ present data in a global prospective observational cohort that height-adjusted total kidney volume (htTKV) is associated with worse kidney outcomes; lower estimated glomerular filtration rate; and for the first time, more patient-important kidney-related complications, including hematuria, hypertension, pain, greater health care utilization, decreased work productivity, and worse patient-reported quality of life (See Figure 1). Growth in the number and size of kidney cysts throughout life, as well as growth in the total kidney size overall, are pathognomonic findings of autosomal dominant polycystic kidney disease (ADPKD). Tolvaptan slows the rate of total kidney volume growth, but regardless of treatment, the kidney irrevocably continues to grow throughout life in all patients with ADPKD. Keeping the musical theme of ADPKD study names following the TEMPO^2^ and REPRISE^3^ trials, OVERTURE represents a momentous achievement with the largest reported cohort of patients with ADPKD, including 3409 participants from 285 study sites in 20 countries!

**Figure 1:**
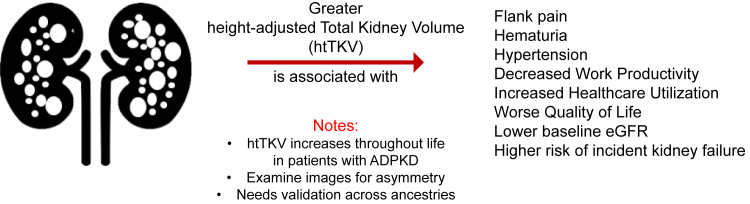
In patients with ADPKD, greater kidney size is associated with negative kidney-related outcomes. In patients with classical symmetric ADPKD appearance, adjustment of height-total adjusted kidney volume (htTKV) for age yields the Mayo Clinic ADPKD Imaging classification. ADPKD, autosomal dominant polycystic kidney disease; eGFR, estimated glomerular filtration rate.

Whereas the REPRISE trial did not include an imaging criterion for inclusion and focused on age-stratified estimated glomerular filtration rate cut-offs in later-stage ADPKD,^3^ the TEMPO trial required participants to have a minimum total kidney volume of 750 ml.^2^ In the end, the mean total kidney volume in TEMPO was far greater at 1.7 L. The development of the Mayo Clinic ADPKD imaging classification was a landmark achievement for risk stratification of patients with ADPKD.^4^ This risk stratification has important ramifications on clinical practice, because those at the highest risk of progression to kidney failure have the most to benefit from treatment. When balancing the pros and cons of treatment, a treatment with a high cost or therapeutic burden may still be worthwhile in the context of high risk and opportunity for significant benefit. The Mayo classification has subsequently been included in clinical practice guidelines from around the world.

In the Mayo classification, patients with symmetric cyst distributions are divided into 5 categories, from slowest kidney growth and lowest risk of kidney failure (class A) to those with fastest kidney growth and highest risk of kidney failure (class E). The classes are based on estimated htTKV growth rates per year of >1.5% (B), >3.0% (C), >4.5% (D), and >6.0% (E). From a single imaging assessment, plotting of an individual’s htTKV (in ml/m) on the y-axis against the age (in years) on the x-axis yields the risk classification and inherently adjusts the htTKV for age. Age is an essential component of htTKV interpretation; for a given htTKV of 1000 ml/m, the Mayo clinic risk stratification can range from B to E depending on the age of the patient at the time of measurement.

In the current article, Perrone *et al.*^1^ assess the association of htTKV directly (unadjusted for age) with ADPKD outcomes. Their models include age as a covariate, which adjusts the likelihood of the outcome based on each participant’s age but does not adjust the htTKV for the expected increase that occurs throughout life. In contrast, that kidney size is associated with patient-important outcomes regardless of age at measurement is a novel finding. Discussion of these technicalities may become moot with the development of more advanced automated imaging-based risk stratification tools based on parenchymal and cyst volume or kidney “texture” beyond measurements of kidney volume.^5^

Patients with asymmetric or atypical cyst distributions (class 2) were excluded from the original Mayo classification^4^; however, they appear to have a favorable prognosis with regard to ADPKD outcomes.^6^^,^^7^ OVERTURE identified fewer than 1% of participants with ADPKD having atypical or class 2 cyst distributions, which is lower than expected compared to the 5% to 10% rate reported in other cohort studies.^4^^,^^6^^,^^7^

Assessment of disease progression with serial imaging remains controversial. Patients and physicians alike desire a measure of disease severity and progression, and to assess the efficacy of a therapeutic plan. However, substantial variability in ellipsoid-based kidney volume measurements occurs as a result of technical artifacts that could lead to misleading conclusions. These challenges led the European Renal Agency working group to specifically recommend against using changes in kidney volume in serial imaging as a marker of disease progression.^8^ OVERTURE had insufficient longitudinal data available to evaluate decline in estimated glomerular filtration rate or changes in htTKV over time.

We need to be cognizant that most published data on ADPKD comes from participants of European ancestry. Population sequencing data suggest that ADPKD should be prevalent at similar frequencies across ancestries.^9^ Nevertheless, TEMPO and REPRISE participants were composed of more than 80% White participants, and OVERTURE similarly has more than 80% White participants. Given the predominantly White participants, the OVERTURE investigators decided to use the 2009 CKD-Epidemiology Collaboration equation, rather than the 2021 “race-free” equation. As pointed out by the authors, a concerted effort is needed improve the diversity of patients in studies of ADPKD to better evaluate the relationship between htTKV and ADPKD outcomes across ancestries. In addition, it will be interesting to assess possible interactions with ancestry-specific risk factors such as *APOL1*.

The OVERTURE investigators have created the largest published prospective cohort of patients with ADPKD, a remarkable achievement. Their work emphasizes the importance of kidney volume assessment in the risk stratification and management of patients with ADPKD. We all look forward to further observations in this large prospective cohort.

## Disclosure

MBL is supported by funding from the Canadian Institutes of Health Research, and has also received compensation from Otsuka, Reata, Bayer, and Sanofi Genzyme as a speaker and advisory board member.
